# Salivary Proteome Changes in Response to Acute Psychological Stress Due to an Oral Exam Simulation in University Students: Effect of an Olfactory Stimulus

**DOI:** 10.3390/ijms22094295

**Published:** 2021-04-21

**Authors:** Lorenzo Zallocco, Laura Giusti, Maurizio Ronci, Andrea Mussini, Marco Trerotola, Maria Rosa Mazzoni, Antonio Lucacchini, Laura Sebastiani

**Affiliations:** 1 Department of Pharmacy, University of Pisa, Via Bonanno 6, 56126 Pisa, Italy; l.zallocco@gmail.com (L.Z.); maria.mazzoni@unipi.it (M.R.M.); 2 School of Pharmacy, University of Camerino, via Gentile III da Varano, 62032 Camerino, Italy; 3 Department of Pharmacy, University G. D’Annunzio of Chieti-Pescara, via dei Vestini, 66100 Chieti, Italy; maurizio.ronci@unich.it; 4 Department of Translational Research and New Technologies in Medicine and Surgery, University of Pisa, 56126 Pisa, Italy; andrea.mussini8@gmail.com (A.M.); laura.sebastiani@unipi.it (L.S.); 5 Laboratory of Cancer Pathology, Center for Advanced Studies and Technology (CAST), University “G. D’Annunzio”, 66100 Chieti, Italy; marco.trerotola@unich.it; 6 Department of Medical, Oral and Biotechnological Sciences, University “G. d’Annunzio”, 66100 Chieti, Italy; 7 Department of Clinical and Experimental Medicine, University of Pisa, via Savi, 56126 Pisa, Italy

**Keywords:** social stress, whole saliva, proteomics, α-amylase, olfactory stimuli, immunoglobulins

## Abstract

The autonomic nervous system (ANS) plays a crucial role both in acute and chronic psychological stress eliciting changes in many local and systemic physiological and biochemical processes. Salivary secretion is also regulated by ANS. In this study, we explored salivary proteome changes produced in thirty-eight University students by a test stress, which simulated an oral exam. Students underwent a relaxation phase followed by the stress test during which an electrocardiogram was recorded. To evaluate the effect of an olfactory stimulus, half of the students were exposed to a pleasant odor diffused in the room throughout the whole session. Saliva samples were collected after the relaxation phase (T0) and the stress test (T1). State anxiety was also evaluated at T0 and T1. Salivary proteins were separated by two-dimensional electrophoresis, and patterns at different times were compared. Spots differentially expressed were trypsin digested and identified by mass spectrometry. Western blot analysis was used to validate proteomic results. Anxiety scores and heart rate changes indicated that the fake exam induced anxiety. Significant changes of α-amylase, polymeric immunoglobulin receptor (PIGR), and immunoglobulin α chain (IGHA) secretion were observed after the stress test was performed in the two conditions. Moreover, the presence of pleasant odor reduced the acute social stress affecting salivary proteome changes. Therefore, saliva proteomic analysis was a useful approach to evaluate the rapid responses associated to an acute stress test also highlighting known biomarkers.

## 1. Introduction

Test anxiety is a form of social anxiety defined as “the set of phenomenological, physiological and behavioral responses that accompany concern about possible negative consequences or failure on an exam or similar evaluative situations” [1]. It is one of the most frequent anxiety forms among students of both sexes and different ages [2,3,4,5]. Test anxiety has been associated with psychological distress symptoms such as sadness, desperation, depression, and distraction and negatively correlated with academic achievements [1,6]. In fact, test anxiety has been reported to affect working memory as well as the retrieval and processing of the learned information [6].

The pattern of cognitive, affective, and physiological responses associated with test anxiety is sustained by the increase of cortisol secretion as well as by the activation of the sympathetic–adrenal–medullary (SAM) axis [7]. Typically, acute mental stress produces marked changes in the concentration of salivary cortisol and proteins. In particular, the levels of salivary α-amylase have been reported to be positively correlated with the acute activation of the sympathetic nervous system, and its secretion has been found to be highly sensitive to acute mental stress as well as test anxiety tasks [8]. The latency between task beginning and salivary α-amylase peak is very short (1–3 min), but such increase persists for about 10 min after task conclusion [9]. Therefore, due to this peculiar secretion time course, salivary α-amylase is considered a useful biomarker of acute stressful events in humans [10]. In addition to α-amylase, other salivary proteins such as chromogranin A (CgA), immunoglobulin A (IgA), immunoglobulin polymeric receptor (PIGR), and cystatin S (CST4) are currently used as salivary stress markers [11].

In the present study, we evaluated the pattern of salivary proteins secretion induced in thirty-eight students by a test anxiety condition, which simulated an oral exam. The proteomic approach, which was used for the first time in test anxiety evaluation, aimed to define at molecular level the global changes of salivary proteins and eventually provide new and early molecular features of test anxiety. Since some essential oils (Eos), such as lavander, peppermint, orange, and bergamot have been reported to have anxiolytic effects [12,13], we also investigated whether diffusion of an Eos odor was able to reduce the test anxiety response and modify related salivary proteomic changes.

## 2. Results

### 2.1. Questionnaires and Heart Rate Results

The scores of the two student groups in the state trait anxiety inventory (STAI) and Leibowitz scales as well as in a battery of questionnaires (Social Phobia Scale (SPS), Social Interaction Anxiety Scale (SIAS), Westside anxiety scale, Social Phobia Inventory (I-SPIN), and subscales of Brief Social Phobia Scale (BSPS)) are shown in Table 1. The scores indicated the absence of social phobia and general low levels of social anxiety. Only the scores of the Westside scale indicated a high-normal level of anxiety for the tests.

ANOVA_RM_ performed on state STAI scores revealed a significant Task effect (F (1.36) = 59.67 *p* = 0.0001) with scores after the stress test (T1) higher (46.26 ± 11.43) than after the relaxation phase (T0) (33.84 ± 8.19). No significant differences between groups were found. Table 2 reports the mean values ± Standard Deviation (±SD) of analyzed heart-beat-intervals (RR)-related parameters in the control and odor groups relative to the different experimental phases.

ANOVA_RM_ performed on cardiac parameters yielded significant Task effects for RR (F (2.58) = 95.87, *p* < 0.000, ε = 0.77), Root Mean Square of Successive Differences (RMSSD) (F (2.58) = 10.25, *p* < 0.001, ε = 0.70), Stress Index (SI) (F (2.58) = 17.10, *p* < 0.000, ε = 0.69). For all parameters, values during the study and presentation phases were significant different from the relaxation period (study vs. relaxation: RR: F (1.29) = 64.76, *p* < 0.000; RMSSD: F(1.29) = 14.61, *p* < 0.001; SI: F(1.29) = 28.79, *p* < 0.000; presentation vs. relaxation: RR: F(1.29) = 132.47, *p* < 0.000; RMSSD: F(1.29) = 11.26, *p* < 0.002; SI: F(1.29) = 20.03, *p* < 0.000). Neither Group nor Group X Task effects were found.

**Figure 1:**
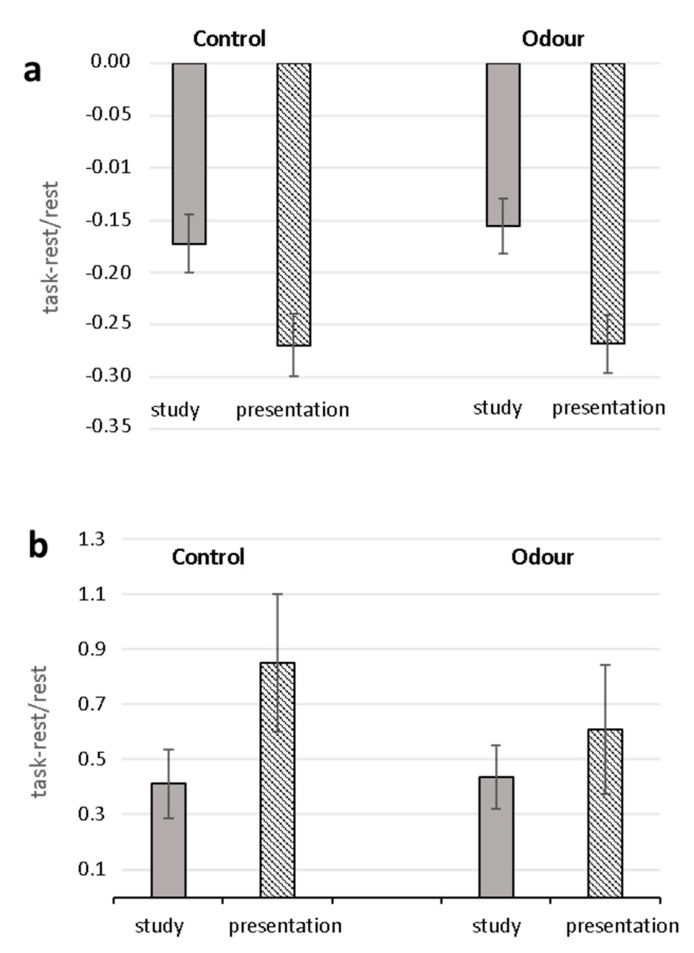
Task-related changes in heart-beat-intervals (RR) (**a**) and Stress Index (**b**) for Control and Odor groups. Mean percentage changes during the study and oral presentation phases with respect to the relaxation condition are shown.

Analysis (ANOVA_RM_) of task-related changes of RR and SI yielded significant Task effects (RR: F (1.28) = 78.42, *p* < 0.0001, ^2^ = 0.737; SI: F (1.28) = 6.50 *p* = 0.017, ^2^ = 0.188) with greater decrease of RR and increase of SI in the oral presentation phase than in the study phase (Figure 1). Neither significant Group effects nor Task X Group interactions were found for both parameters. No significant effects were found for RMSSD.

The mean scores (±SD) of the five Speech Preparation Questionnaire (PREP) items in the two experimental groups are shown in Table 3. Analysis of PREP scores did not yield any significant difference between the two groups in any of the items.

### 2.2. Comparative Proteomic Analysis and Validation

**Figure 2:**
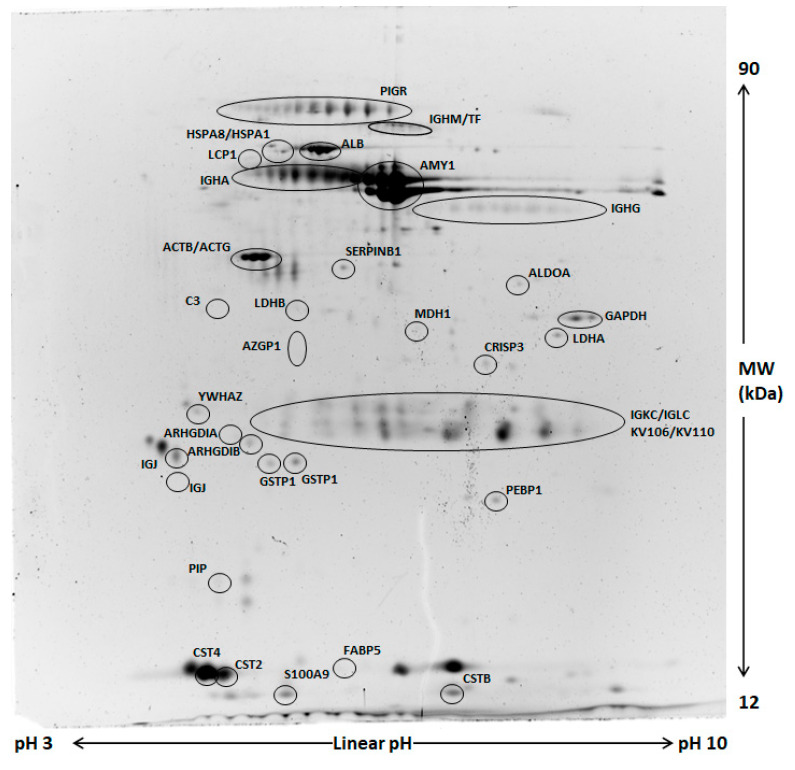
Representative two-dimensional electrophoresis (2DE) of whole saliva (WS). Two-hundred µg of proteins were separated by 2DE using 18 cm pH 3–10 linear strip and 12% SDS-PAGE. Gels were stained by fluorescent dye. All circled spots indicate proteins identified by nano-LC-ESI MS/MS with gene names also reported in Table 3 and Table 4.

Comparative analysis of whole saliva (WS) two-dimensional electrophoresis (2DE) images was performed in each group (control and odor) between samples collected at T1 and those obtained at T0. A representative image of WS protein extracts is shown in Figure 2. A significant change of salivary profiles was observed after the anxiety test both in the control and odor group. After computational comparison of images, a total of 64 and 28 protein spots were found to be differentially expressed in the control and odor group, respectively. These protein spots were chosen for excision and identified by nanoLC-ESI-MS/MS analysis. The list of identified proteins, which also include the respective molecular weight (MW), isoelectric point (pI), coverage values of nanoLC-ESI-MS/MS, ratios, and their relative p-values is shown in Table 4 and Table 5 for the control and odour group, respectively. Overall, α-amylase expression increased, while PIGR expression decreased in both groups at T1 compared to T0. Interestingly, 2DE results suggested a minor increase of two main common spots of secretory α-amylase in the odor group with respect to the control one. However, these findings were not subsequently confirmed by Western blot (WB) analysis, suggesting the difference can concern only specific isoforms of α-amylase [14]. Moreover, a significant reduction of immunoglobulins was generally detected even though different chains were characteristically deregulated in control and odor group. In addition to α-amylase and immunoglobulin chains, an exclusive set of salivary proteins resulted in deregulation in the control group after the anxiety test, namely cystatin SA (CST2) and S, cysteine-rich secretory protein 3 (CRISP3), plastin-2 (LCP1), zinc-alpha-2-glycoprotein (AZGP1), leukocyte elastase inhibitor (SERPINB1), and Rho GDP-dissociation inhibitor 2 (ARHGDIB). Moreover, a significant expression change of several metabolic enzymes (lactate dehydrogenase A (LDHA), malate dehydrogenase (MDH1), and glyceraldehyde-3-phosphate dehydrogenase (GAPDH)) was observed in the control group at T1 with respect to T0. Of note, in salivary samples of the odor group, a peculiar deregulation of fatty acid-binding protein 5 (FABP5), phosphatidylethanolamine-binding protein 1 (FEBP1), and cystatin-B (CSTB) was detected.

**Figure 3:**
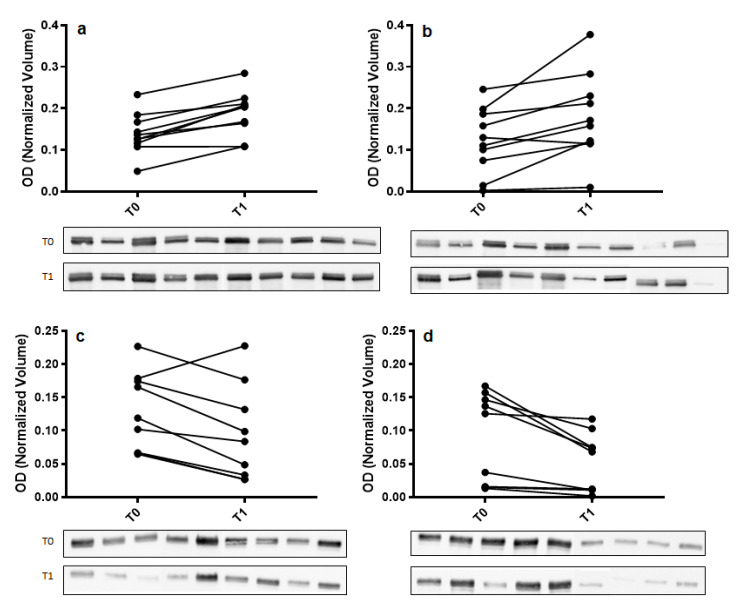
Validation of α-amylase (panel (**a**,**b**)) and immunoglobulin α chain (IGHA) (panel (**c**,**d**)) in whole saliva (WS) samples at T0 (after relaxation phase) and T1 (after anxiety test) from control (panel (**a**,**c**)) and odor exposed (panel (**b**,**d**)) subjects using Western blot (WB) analysis. A graphical representation of normalized optical density (OD) of α-amylase and IGHA bands is shown. Each pair of connected points represents one experiment using T0 and T1 WS samples obtained from a single subject. The *p*-values were determined by paired t-test. Representative blots are shown below the graphs. A single immunoreactive band with apparent molecular weight approximately of 55 kDa and 67 kDa was obtained for α-amylase and IGHA, respectively.

To confirm 2DE results, Western blot analysis with specific antibodies was used to validate the expression change of α-amylase and immunoglobulin α chain (IGHA). For each tested protein, the optical density (OD) of specific immunoreactive bands was normalized with total protein OD. A single immunoreactive band of apparent molecular weight of 55 kDa and 67 kDa was detected for α-amylase and IGHA, respectively. Figure 3 shows a graphical representation of normalized OD of α-amylase and IGHA obtained for each subject at T0 and T1 in control and odor groups.

Concerning the effect of odor on WS protein profiles at T0, only a few significant changes were observed unconnected with the stress effect (data not shown). For example, according to a previous reported observation, an increase of prolactine inducible protein (PIP) expression was detectable in samples from odor-exposed students with respect to controls [15].

### 2.3. Ingenuity Pathways Analysis (IPA)

**Figure 4:**
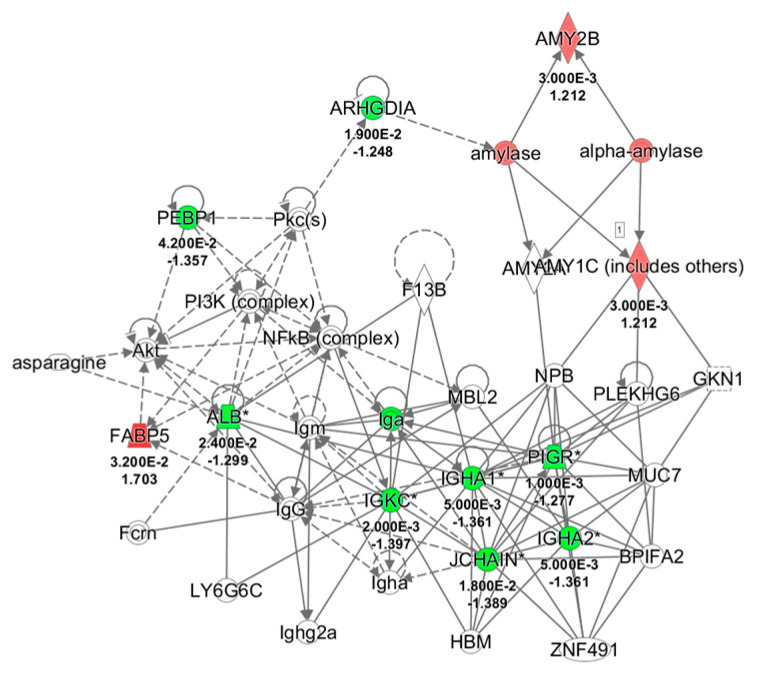
Network analysis of whole saliva (WS) differentially expressed proteins obtained from T1 versus T0 comparison in odor group using Ingenuity Pathway Analysis (IPA) software. The network shows proteins interactions in the context of “Humoral Immune response and Inflammatory response” along with corresponding protein-to-protein direct (solid line) or indirect (dashed line) interactions based on published literature information. IGHA1/IGHA2, Immunoglobulin (Ig) α chain C, Iso 1, 2; PIGR, Polymeric immunoglobulin receptor; IGKC, Ig κ chain C region; SERPINB1, Leukocyte elastase inhibitor; ALB, serum albumin; JCHAIN, Immunoglobulin J chain; ARHGDIA, Rho GDP-dissociation inhibitor 1; AMY1A/1B, α-amylase, 1A/1B; FABP5, Fatty acid-binding protein, epidermal; PEBP1, Phosphatidylethanolamine-binding protein 1; Pkc, Protein kinase C; NFkB, Nuclear factor NF-kappa-B; Akt, RAC-serine/threonine-protein kinases; NPB, Neuropeptide B; GKN1, Gastrokine-1; PLEKHG6, Pleckstrin homology domain-containing family G member 6; MUC7, Mucin-7; BPIFA2, BPI fold-containing family A member 2; ZNF491, Zinc finger protein 491; HBM, Hemoglobin subunit mu; MBL2, Mannose-binding protein C; LY6G6C, Lymphocyte antigen 6 complex locus protein G6c; Fcrn, IgG receptor FcRn large subunit p51; F13B, Coagulation factor XIII B chain; PI3K, Phosphatidylinositol 3-kinase.

All differentially expressed proteins, related to each group, were included in the bioinformatics analysis to recognize their molecular and cellular functions and highlight the interactions inside a specific network. Twenty-nine and eleven of the deregulated proteins resulting respectively from control and odor groups, were associated to the same network the “Humoral Immune response and Inflammatory response” with a score of 40 and 30, respectively (Figure 4). Moreover, comparison of IPA for protein changes of control and odor groups was performed, and the top ten upstream regulators whose activity changed in a significant manner are listed in Table 6. In particular, interleukin 4 (IL4), interleukin 1β (IL1β), and lipopolysaccharide (LPS) were predicted inhibited (z-score values < −2) in the control group. On the other hand, an activation of lysine-specific histone demethylase 1A (LSD1A or KDM1A) was suggested by z-score value = 2.

## 3. Discussion

In the present study, we evaluated the pattern of salivary protein secretion induced in University students by a test anxiety condition, which simulated an “oral exam” and explored whether pleasant odors diffused in the room were able to reduce test anxiety by modulating both participant subjective experience and physiological responses, namely increased heart rate and the secretion of anxiety-related salivary proteins.

Reports on state anxiety indicated that the fake exam was effective at inducing test anxiety. In fact, before the oral presentation, all participants reported high levels of nervousness associated with low expectations regarding their performance outcome, and at the end of the test session, they all reported higher levels of perceived anxiety with respect to the post-relaxation period. Moreover, the subjective experience of participants was associated with a reduction of the cardiac indices of vagal activity (RR, RMSSD) and an increase of SI, which indicates a reduction of heart rate variability (HRV) considered a marker of increased sympathetic activity [16]. Both the subjective and cardiovascular responses were not modulated by the presence of the olfactory stimulus, being similar in the presence or absence of the pleasant odor.

Proteomic analysis of salivary proteins unveiled a different pattern of responses after the test anxiety task in control and odor groups, with the exception of α-amylase, PIGR, and IgA, which showed comparable expression changes. No significant changes of protein expression were observed after the relaxation phase.

In both experimental conditions, the stress test induced an increase of expression of α-amylase expression, whereas a decrease of PIGR and immunoglobulins (Igα, Igγ, and Igµ-heavy chains, κ and λ light chains) was found.

Salivary α-amylase is an enzyme produced in the oral cavity by salivary glands, mainly the parotid gland. Its primary function is to hydrolyze polysaccharides into lower molecular weight carbohydrates, but it also seems to be involved in maintaining mucosal immunity (e.g., inhibition of streptococcus propagation/colonization) [17]. Sympathetic nerves induce α-amylase secretion via noradrenaline release. In fact, pharmacological tasks have revealed that salivary α-amylase levels are increased and suppressed by the β-adrenergic agonists [18,19] and antagonists applications, respectively [19,20,21,22]. Salivary α-amylase is also considered a marker of sympathetic autonomic nervous system (ANS) activity during acute and chronic stress [23,24,25,26,27]. The psychophysiological evidence is supported by pharmacological results showing inhibition of stress-related increase in salivary α-amylase by the adrenergic blocker propranolol [22].

The change in α-amylase secretion we found after the exam simulation is in line with previous findings, which indicate a rapid increase of this protein in response to acute laboratory stress procedures [12,26,28]. A similar increase of α-amylase has been also described previously in health care professionals, army nurses, and police officers performing standardized pre-hospital emergency simulations [27], combat casualty stress scenarios [24], and reality-based school shooting simulations, respectively [23,25]. Moreover, previous studies have reported positive correlations of stress-induced salivary α-amylase levels with increases of heart rate and negative correlations with decreases in heart rate variability (RMSSD) [29,30,31]. In the present study, the increase in α-amylase parallels the RR and HRV changes. However, no significant correlations were found.

Overall, our observations suggest that salivary α-amylase is a sensitive biomarker of test anxiety, particularly in acute situation where evaluation of cortisol levels, whose peak secretion takes at least 20 min to occur, are less consistent [32] and controversial [25,27]. Moreover, the difference of increase observed in the presence of olfactory stimulus suggests that the presence of essential oils could modulate the ANS salivary secretion of different proteoforms of α-amylase [14].

Concerning IgA, our findings showed a post task decrease of both the Igα chains isotypes, IGHA1 and IGHA2. This finding contrasts with results of most previous studies, which report an increase of salivary IgA concentration after an acute stress [26,30,31,33,34,35,36,37,38]. However, some studies have also highlighted that stress-related IgA secretion is highly influenced by both the experimental protocol and psychological participants’ characteristics. For instance, an increase and decrease of IgA secretion have been found in response to active (cognitive tasks) and passive (passive viewing of surgery) task conditions, respectively [39]. Campisi et al. [28] have shown a trend of increased salivary IgA levels in response to the Trier Social Stress Test (TSST) but devoid of any statistical significance. Finally, IgA decreases have been reported during a cold pressor task [40] and in response to dental surgery [41]. Our experimental protocol was a simulation of an exam, and its peculiarity was its very short duration, which was much shorter than that of previous studies. For instance, in the studies employing the TSST, which includes a free speech and a mental arithmetic task, the session lasts about 15 min, while in another study, it lasted about one hour [17,26,32].

Salivary IgA are secreted by three pairs of major glands (parotid, sublingual, and submandibular glands) and minor accessory glands in the tongue, lip, and palate [42]. Differently from most glands, which have sympathetic and parasympathetic innervation, the sublingual glands and some of the accessory glands, which are responsible for secreting the larger part of salivary IgA, receive a parasympathetic input only [43,44]. Previous neurophysiological studies have shown that stimulation of the parasympathetic nerves causes an increase of secretory IgA [45,46]. The parasympathetic peptides substance P and VIP also seem to increase IgA secretion [47,48,49,50]. Thus, the different IgA response associated to different social anxiety conditions could suggest that each condition modulates the secretion of salivary glands differentially. In our case, the IgA decrease could be sustained by a reduction of sublingual and accessory gland secretion related to parasympathetic withdrawal. In addition, it is worth noting that IgA secretion is not proportional to the activity of autonomic nerves innervating the salivary glands, meaning that in our test conditions, the parasympathetic withdrawal could have elicited a stronger effect on IgA secretion than the sympathetic activation.

The decreased secretion of IGHA was associated to a parallel decrease of PIGR, thus suggesting that the secretion decrease is likely due to a reduction of the glandular transport capacity rather than to a reduced release of IgA from B-lymphocytes [17]. In fact, salivary IGHA and PIGR are indicators of IgA production and glandular transport capacity, respectively, and their concomitant measure allows estimating whether stress-related IGHA reduction is attributable to a decrease of IgA release by B-lymphocytes or a decreased transport capability.

In the group with the olfactory stimulation, a reduction of IgA secretion similar to that of the control group was found. However, in contrast with controls, no decrease of IgG and IgM was found.

In the control group, CST2 and CST4 were also increased. CST2 and CST4 belong to the cystatin superfamily, which prevents cell death caused by virus replication and bacterial invasion by inhibiting cysteine proteases. Our findings are in line with previous studies, which showed an increase of CST2 and CST4 after stress [17,26,38] and identified CST4 as a potential marker of acute stress. In the odor group, no change in CST2 and CST4 was found, while a decrease of CSTB occurred. CSTB is an endogenous cathepsin inhibitor localized in different cell types and extracellular fluids, too [51,52,53]. From a functional point of view, CSTB has been associated to the macrophage activation, apoptosis prevention [54], regulation of cell cycle entry [55], protection against oxidative stress of mitochondria [56] and neurons [57] due to its neuroprotective role, and it is considered part of the innate immunity system. However, to date, the exact function of salivary CSTB is still unclear [58].

In the control group, other proteins involved in oxidative and immune processes such as glutathione S-transferase P (GSTP), PIP, calgranulin B (S100-A9), and GAPDH decreased in response to the stress test. All these proteins have a role in mucosal immunity including anti-inflammatory effects and inhibition of bacterial growth and colonization of the mouth [59,60]. In particular, increases of GSTP and PIP have been reported by previous acute stress studies including psychosocial stress tests [26]. GSTP is an enzyme that prevents oxidative stress-dependent cell death of mucosal epithelia, thus reducing the risk for infection [61]. PIP is also involved in immune regulation in the mouth [62] and has been found to inhibit the growth of many bacterial strains by binding to their surface. Our results indicating a general decrease of mouth mucous membrane defenses contrast with previous findings and suggest that the response to social stress is strictly dependent on specific features of the stressor and/or stressful context. This hypothesis is supported by our findings in the group with the olfactory stimulation. In fact, the presence of the pleasant odor prevented the decrease of these proteins, thus exerting a sort of protection against the negative effects of the test anxiety on upper airway immunity.

Although the test anxiety caused deregulation of more salivary proteins in the control group than in the odor one, in the former, only α-amylase was increased, while α-amylase and FABP5 were present in higher amounts in the latter. According to previous studies [63], FABP5 is considered involved in oral fatty acids perception. Thus, on the basis of the relevant role of olfaction in taste perception, we can assume that the increased secretion of FABP5 was likely more related to the odor of the essential oil itself, whose components also include fatty acids than to the stressor.

IPA revealed that most of the deregulated proteins both in the control and odor group were associated to the same network that is the “Humoral Immune and Inflammatory Responses”. Moreover, comparison between the two groups indicated significant difference in many of the predicted upstream regulators. In particular, two cytokines (IL4 and IL1β) and LPS were predicted inhibited (z-score values < −2) in the control group, whereas no changes of these regulators were predicted in the odor group. The effect of stressors on interleukins levels has been extensively studied and depend on the type and timing of stress [64]. In fact, activation or inhibition of specific interleukins underlines a proinflammatory or anti-inflammatory response. Interestingly, in the control group, KDM1A activation is suggested by the high z-score. KDM1A, also known as lysine-specific-demethylase 1 (LSD1), is an epigenetic enzyme that plays a role as a molecular transducer of stressful stimuli as well as a stress-response modifier [65,66]. In fact, psychosocial stress produces an acute increase of LSD1 expression both at the transcriptional and splicing levels, suggesting that LSD1 is involved in the adaptive response to stress. In particular, LSD1 and its splicing variant neuroLSD1 act as negative and positive modulators of activity of IEGs egr1 and c-fos in mice [65], respectively. NeuroLSD1 mutant KO mice fail to transduce stressful stimuli into proper anxiety-related plasticity [65]. The neuroLSD1/LSD1 ratio is high in neurons of young mice and reaches a steady state in adult neurons. It has been hypothesized that physiological neuroLSD1 decrease under stressful conditions could represent a molecular mechanism concurring in stress resilience.

Intriguingly, our experimental protocol has examined a young population, which can be also potentially susceptible to a positive modulation of neuroLSD1 in response to psychosocial stress. This is a very fascinating point, which requires further investigations since it suggests the presence of an epigenetic control in the molecular translation of stress stimuli in a very short time.

In conclusion, the test anxiety is a multi-dimensional construct characterized by the involvement of different systems that can be monitored by measuring specific markers at the right time. The physiological response to a test anxiety situation depends both on specific features of the task and contextual factors. Our findings indicate that pleasant odors differentially modulate the anxiety-related responses induced by an exam-like condition, being effective only on the autonomic component, which controls the salivary secretion. Moreover, the occurrence of inattentional smell blindness in the anxiety test condition suggests that odors can exert their effects on autonomic functions when they are not consciously perceived, too [67].

A limitation of the study is that possible gender-related differences in stress responses could not be analyzed due to the small sample size. In fact, hormone-related differences are likely to occur in social anxiety. Further studies will be performed in order to address this point.

## 4. Materials and Methods

### 4.1. Participants

The study was performed in accordance with the ethical standards of the Declaration of Helsinki and approved by the Committee on Bioethics of the University of Pisa (Review No. 5/2018, 30th November 2018). All participants read and signed an informed consent.

The sample size was determined according to a priori power analysis performed with G * Power 3.1.9.7. With α = 0.05, power = 0.90, and a moderate effect size = 0.3, the required sample size for Repeated Measures ANOVA, with 2 groups and 2 measurements was 32 while with 2 groups and 3 measurements was 26. Our total sample size was 38. Participants were healthy young adults (21 females and 17 males; mean age ± SD, 24.9 ± 2.3; mean BMI ± SD, 21.5 ± 2.4) were recruited among the students of the University of Pisa. Participants were separated in two groups, control and odor, each of 19 students (control: 10F/9M, mean age ± SD, 23.8 ± 2.1, mean BMI ± SD 21.3 ± 1.9; odor: 9F/10M, mean age ± SD, 23.6 ± 2.4, mean BMI ± SD 21.6 ± 2.1)

Inclusion criteria required that participants had no history of medical, neurological, or psychiatric disorders; no systemic diseases; trait questionnaire for trait anxiety (STAI-Y2) and Liebowitz Social Anxiety Scale [68] scores below 45 and 58, respectively; no experience in meditation and/or relaxation techniques; scores above 114 in the Odor Awareness Scale [69], which allows the evaluation of olfactory sensibility, the impact of pleasant/unpleasant odors on mood, and the capability to perceive the odors during different conditions. Finally, no dental/periodontal diseases had required: specifically, all the recruited participants had undergone a screening for dental/periodontal disease with a basic periodontal examination within 15 days prior to the test. Only students whose results showed no bleeding after probing, no pocketing, no dental caries, and no wound and lesions of the oral mucosa were included in the study.

### 4.2. Physiological Parameters

During the whole experimental session, we recorded the electrocardiogram (ECG). The ECG was recorded by means of 3 Ag/AgCl disposable electrodes placed on the chest according to the standard DII lead and connected to the PSYLAB SAM amplifier (Contact Precision Instruments). ECG was acquired at 1000 Hz and band pass filtered (0.3–30 Hz). ECG signals were analyzed by means of Kubios HRV software in order to obtain the series of RR and HRV measures in the time and frequency domain.

### 4.3. Experimental Protocol

Experiments were scheduled at 10:30 a.m. Participants were instructed to abstain from eating, drinking coffee or juices, smoking, chewing gums or candies, tooth brushing, and using lipstick for at least 3 h before the beginning of the experimental session. Participants were also invited to fill in (online by means of Google Forms) a battery of questionnaires aimed at evaluating social anxiety and phobia: the SPS, the SIAS [70], the Westside anxiety scale, the I-SPIN [71], and the BSPS [72]. For all questionnaires, we used validated Italian versions. At his/her arrival, the participant read and signed the informed consent and was briefly informed on the experimental procedures. Then, she/he was equipped with the ECG recording electrodes and invited to sit in a comfortable armchair. ECG was recorded throughout the whole experimental session.

Participants were randomly assigned to the control or odor group, and before the beginning of the experimental session, the odor group participants were asked to identify 4 different essential oils (Eos): orange (citrus aurantium), mint (mentha piperita), lavender (lavandula hybrid), and bergamot (citrus bergamia) (Flora srl, Pisa, Italy). Orange and bergamot were obtained by means of cold press extraction, while mint and lavender were obtained by steam distillation of the whole flowered plant and flowers, respectively.

Participants were required to indicate which Eos they considered the most pleasant. Then, the favorite fragrance was diluted in 250 mL of distilled water (solution 0.05–0.06%) and diffused in the room during the different phases of the experimental session (relaxation, study phase, and oral presentation) by means of a diffusing system (Avaspot XFFR-XXJ-003). In order to avoid habituation effects, the diffusing system vaporized puffs of the fragrance every 30 s. The diffusing system was positioned at about 2 m from the participant’s head. Participants of the odor group were informed that the chosen Eo would be diffused in the room during the relaxation phase to facilitate it. In contrast, in order to avoid possible expectancy-related and/or placebo effects of pleasant odors on the autonomic and immune system during the test [73], participants were not informed that the Eo would be diffused in the room also during the successive experimental phases. Indeed, in the final interview, all participants reported that during relaxation, they were aware of the odor and its presence had made the relaxation experience more pleasant, while none of them could say without a doubt whether an odor was diffused in the room during the study and presentation phases.

The experimental session consisted of 3 phases: relaxation, study, and oral presentation.

Relaxation (10 min): the participant was asked to close his/her eyes and try to relax, breathing at her/his usual pace. During this phase, the participant rested on a semi-reclined armchair while listening to the recorded sound of sea waves through headphones. At the end of the relaxation phase, the participant completed the STAI Y2 questionnaire upon state anxiety and supplied a sample of saliva.

Study phase (3 min): the participant was seated in front of a computer screen where a written text was presented for 3 min. The participant was asked to read and memorize as much information as possible in the short period of time. The text was chosen so that the included information did not belong to the field of study of the participant. At the end of the study phase, the participant completed the PREP questionnaire, a 5-item self-report questionnaire assessing, on a Likert scale 0–5 (0 = extremely low; 5 = very high), the confidence, nervousness, calmness, and preparedness of an individual before he/she gives the oral presentation. Participants were also required to “predict” the goodness of their performance on a Likert scale 0–5 (0 = very bad; 5 = very good).

Oral presentation (2 min): the participant was seated in front of a professor and was asked to orally expose the studied text. The participant was informed that the LS professor was designated to evaluate, on a 30-point scale (the same used by the Italian university to score exams), his/her performance in terms of remembered information, accuracy in reporting details, and verbal fluency. The voice of the participant was recorded by means of the software Audacity, and the participant was informed that the recorded audio would be listened to by a group of students involved in a program organized by the University of Pisa aimed at the improvement of oral exams performance. A timer on the screen marked the passing of time. If the participant stopped talking before the end of the fixed time for a period longer than 5 sec or declared that he/she did not remember further information, the session was interrupted. At the end of the presentation phase, the participant completed the state STAI Y2 questionnaire and supplied a sample of saliva.

Then, the experimenter asked the participant whether he/she had perceived any aroma and in which phases of the experimental session. Finally, the participant was informed about the real goal of the study and that no committee of professors would actually be listening to her/his recorded voice.

### 4.4. Questionnaires and Heart Analysis

STAI Y2 scores obtained in the two groups before (T0, at the end of the relaxation) and after the anxiety test (T1) were compared by means of repeated measures ANOVA (ANOVARM) with Task (T0, T1) as the within-subjects factor and group (odor, control) as the between-subjects factor.

The scores obtained by the two groups on the five self-rating items of the PREP were compared by means of a chi-squared Pearson test.

In order to evaluate task-related changes in heart activity, we used a few measures of HRV in the time and frequency domain. Namely, the series RR, the RMSSD, and the high-frequency band (HF) were used to evaluate vagal activity. The modified version of Baevsky Stress Index (SI) [16] supplied by the Kubios software was obtained from the RR series and employed as a measure of sympathetic activation. As a result of technical problems, some heart recordings could not be included in the analysis. Thus, the final sample for cardiac parameters consisted of 31 participants (controls, 14; odor, 17). For each participant, we calculated the mean value of RR, RMSSD, and SI during relaxation, study, and oral presentation phases. In order to study the effects of the odor throughout the experimental session, we performed separate ANOVARM on RR, RMSSD, and SI mean values with Task (relaxation, study, oral presentation) as the within-subject factor and group (odor, control) as the between-subjects factor.

Moreover, in order to compare the task-related changes of the two groups during each task, for RR, RMSSD, and SI values, we calculated the percentage changes (study and oral presentation) with respect to the relaxation condition (% change = (task -relaxation)/relaxation * 100) and applied, separately for each variable, ANOVARM with Task (study, oral presentation) as the within-subjects factor, and group (odor, control) as the between-subjects factor. The normality of distributions and homogeneity of variance were checked using Kolmogorov–Smirnov and Levine tests, respectively. Greenhouse–Geisser correction for non-sphericity was applied when necessary. For analysis, percentage changes were log transformed. For all tests, significance was set at *p* < 0.05. Data were analyzed using IBM SPSS Statistics.

### 4.5. Salivary Samples

Unstimulated WS collection and processing was performed essentially as previous described [74]. WS samples were collected with Salivettes (Sarstedt Inc., Newton, MA, USA), which allow the extraction of saliva by means of a cotton swab (without citric acid) placed in the mouth. Participants were instructed to move the swab through their mouth using their tongue for 1 min without biting, chewing, and touching it with their hands. The swab soaked with saliva was collected into a Falcon tube and centrifuged (1000× *g* for 2 min). Then, the WS sample was transferred into an Eppendorf and centrifuged at 17,000× *g* for 20 min, at 4 °C. The surnatant (about 1 mL of WS) was stored at −80 °C. In order to minimize protein degradation, samples were processed immediately and kept on ice during the process. Protein amount was determined using the Bio-Rad DC-protein assay. The mean value of WS protein concentration was 2.78 ± 0.13 mg/mL.

### 4.6. Proteomic Analysis

2DE was essentially performed according to Ciregia et al. [75]. Briefly, 200 µg of proteins were filled up to 350 µl in rehydration solution added with 1% IPG buffer pH 3–10 L and 0.8% pharmalyte. Immobiline Dry-Strips 18 cm, linear gradient pH 3–10, were rehydrated overnight in the sample and then transferred to the Ettan IPGphor II (GE Health Care Europe; Uppsala, Sweden) for isoelectrofocusing (IEF). The second dimension (SDS-PAGE) was carried out by transferring the proteins to 12.5% polyacrylamide gels, and then, gels were stained with 1 µM Ruthenium II tris (bathophenanthroline disulfonate) tetrasodium salt (RuBPS) (Cyanagen, Bologna, Italy) [76]. Images were acquired using ImageQuant LAS4010 (GE Health Care) and analyzed using Same Spot (V4.1, Total Lab, Newcastle Upon Tyne, UK) software as previously described [75]. Comparative analysis was performed for each group, between two experimental conditions (before and after the anxiety test). The significance of the differences of the normalized volume for each spot was calculated by paired ANOVA test. Therefore, the protein spots of interest were selected and cut out from the gel for identification by LC-MS/MS.

### 4.7. In-Gel Digestion and Mass Spectrometry

The gel pieces were digested as reported by Giusti et al. 2018 [77]. Samples were analyzed by LC-MS/MS as previously described [78] using a Proxeon EASY-nLCII (Thermo Fisher Scientific, Milan, Italy) chromatographic system coupled to a Maxis HD UHR-TOF (Bruker Daltonics GmbH, Bremen, Germany) mass spectrometer. Briefly, peptides were loaded on the EASY-Column C18 trapping column (2 cm L., 100 µm I.D., 5 µm ps, Thermo Fisher Scientific) and then separated on an Acclaim PepMap100 C18 (25 cm L., 75 µm I.D., 5 µm ps, Thermo Fisher Scientific) nanoscale chromatographic column at a flow rate of 300 nL/min and with a standard gradient from 3 to 35% of acetonitrile in 15′. The mass spectrometer was equipped with a nanoESI spray source and operated in positive ion polarity and Auto MS/MS mode (Data Dependent Acquisition—DDA), using N2 as collision gas for CID fragmentation. In-source reference lock mass (1221.9906 *m*/*z*) was acquired online throughout the runs.

Raw data were processed with DataAnalysis v. 4.2 to apply the lock mass calibration and then loaded in PEAKS Studio v7.5 software (Bioinformatic Solutions Inc., Waterloo, ON, Canada) using the ‘correct precursor only’ option. The mass lists were searched against the NextProt database (downloaded December 2018 and containing 42,184 entries). Carbamidomethylation of cysteines was selected as fixed modification and oxidation of methionines, deamidation of asparagine and glutamine, and acetylation of lysines and at N-terminus were set as variable modifications. Non-specific cleavage was allowed to one end of the peptides, with a maximum of 2 missed cleavages and 2 variable PTMs per peptide. Ten ppm and 0.05 Da were set as the highest error mass tolerances for precursors and fragments, respectively, and the -10lgP threshold for PSMs was manually set to 35.

### 4.8. WB Analysis

WB analysis was performed as previously described [76] for immunoglobulin alpha chain C and α-amylase. Briefly, sample aliquots of WS were mixed with the Laemmli solution, run in 8–16% polyacrylamide gels (Mini-PROTEAN^®^ TGX Precast Gels, Biorad, Hercules, CA, USA) using a mini-Protean Tetracell (Biorad), and transferred onto nitrocellulose membranes (0.2 µm) using a Trans-Blot Turbo transfer system (Biorad). The amount of proteins loaded and the dilution of primary antibody (IGHA1: Thermo scientific—PA5-14361, α-amylase: Cell signaling—#3796) was different depending on each analyzed protein (IGHA1:5 μg of proteins and dilution of 1:500, α-amylase: 3 μg and a dilution of 1:1000). The immunocomplexes were detected using a peroxidase-labeled secondary antibody (goat-anti rabbit IgG, dilution 1:10,000, Enzo life sciences #ADI-SAB-300). Immunoblots were developed using the ECL detection system. The chemiluminescent images were acquired by LAS4010 (GE Health Care). The immunoreactive specific bands were quantified using Image Quant-L software. In order to normalize the optical density (OD) of immunoreactive bands, the optical density of total proteins was calculated. Therefore, immediately after the electroblot, membranes were stained with 1 µM RuBPS [79].

Statistical analysis was carried out with paired Student t-test making a comparison of protein expression levels before (at the end of the relaxation) and after the anxiety test for two groups (odor, control).

Data were analyzed using IBM SPSS Statistics Version 19. *P* < 0.05 was considered statistically significant.

### 4.9. IPA

Proteins found differentially expressed in each group were functionally analyzed using the Ingenuity Pathway Analysis (IPA, QIAGEN Redwood City, CA, USA, www.qiagen.com/ingenuity (accessed on 20 April 2021), Build version: 321501M Content version: 21249400) with the aim to determine the predominant canonical pathways and interaction network involved. The network proteins associated with biological functions and/or diseases in the Ingenuity Pathways Knowledge Base were considered for the analysis. The created genetic networks describe functional relationships among proteins based on known associations in the literature. A comparison of the different analyses was created, and the upstream regulators whose activity appears to change in a significant manner according to the activation z-score value were shown.

## Figures and Tables

**Table 1:** Questionnaire scores.

Scales	Control	Odour
	mean ± SD	mean ± SD
Liebowitz	41.89 ± 16.76	38.89 ± 16.66
Westside	2.81 ± 0.76	2.76 ± 0.59
I-SPIN	21.11 ± 12.12	20.83 ± 8.26
BSPS-Fear	6.21 ± 3.94	7.06 ± 2.76
BSPS-Avoidance	6.21 ± 3.58	6.28 ± 3.16
BSPS-Physiology	4.53 ± 2.61	4.89 ± 3.28
STAI-Y2	50.32 ± 11.94	48.50 ± 9.71
SPS	19.21 ± 13.16	20.50 ± 8.57
SIAS	25.84 ± 13.89	23.94 ± 10.09

I-SPIN, Social Phobia Inventory; BSPS, Brief Social Phobia Scale; STAI-Y2, trait anxiety inventory for trait anxiety; SPS, Social Phobia Scale; SIAS, Social Interaction Anxiety Scale.

**Table 2:** Heart rate scores.

Parameters	Phases	Control	Odour
		mean ± SD	mean ± SD
	relaxation	866.22 ± 88.12	910.07 ± 98.38
RR (msec)	study	718.11 ± 119.49	767.92 ± 129.23
	presentation	631.52 ± 96.10	671.09 ± 123.21
	relaxation	51.86 ± 23.69	51.65 ± 27.83
RMSSD (msec)	study	41.95 ± 18.85	38.24 ± 23.67
	presentation	37.32 ± 22.31	36.38 ± 26.31
	relaxation	8.15 ± 2.34	8.88 ± 3.08
Stress Index	study	11.23 ± 3.43	12.13 ± 4.12
	presentation	14.38 ± 7.27	13.09 ± 5.58

RR, heart-beat-intervals; RMSSD, Root Mean Square of Successive Differences.

**Table 3:** Speech Preparation Questionnaire (PREP) scores.

PREP	Control	Odour
	mean ± SD	mean ± SD
confidence	2.43 ± 1.02	2.35 ± 1.05
nervousness	2.86 ± 0.77	2.88 ± 1.05
calmness	2.42 ± 0.94	2.82 ± 1.07
preparedness	2.43 ± 0.75	2.23 ± 1.03
how good	2.14 ± 0.53	1.94 ± 0.82

**Table 4:** List of identified salivary proteins found differentially expressed after the anxiety test in the control group.

#	ID	Protein	Gene	Cov	Pep	Unic	MW (th)	pI (th)	*p*-Value (min–max)	Ratio (T1/T0) (min–max)
67, 69	P01833	Polymeric immunoglobulin receptor	*PIGR*	24	18	18	83,284	5.59	0.030–0.044	0.80–0.86
98, 100, 102, 105	P01871	Ig µ chain C region	*IGHM*	33	15	15	49,307	6.35	0.008–0.046	0.75–0.80
98, 100, 102, 105	P02787	Serotransferrin	*TF*	16	12	12	77,064	6.7	0.008–0.046	0.75–0.80
126, 128	P11142	Heat shock cognate 71 kDa protein	*HSPA8*	45	30	28	70,898	5.37	0.003–0.006	0.69-0.77
170 187	P02768	Serum albumin	*ALB*	74	61	61	69,367	5.67	0.008	0.66–0.71
186	P0DMV8 P0DMV9	Heat shock 70 kDa protein 1A, 1B	*HSPA1A HSPA1B*	37	25	20	69,921	5.48	0.0176	0.67
211	P13796	Plastin-2	*LCP1*	29	16	16	70,289	5.29	0.0185	0.566
271, 339, 348	P0DUB6	α-amylase,1A	*AMY1A*	76	99	99	57,768	6.34	0.0001–0.004	1.30–1.39
364, 368, 369, 370, 373, 374, 375, 376, 1148	P01857 P01859	Ig γ-1, 2 chain C region	*IGHG1*, *IGHG2*	21	7	5	36,106	8.46	0.0001–0.012	0.52–0.80
457, 461, 464	P60709 P63261	Actin, cytoplasmic Iso 1, 2	*ACTB*, *ACTG*	29	10	9	41,737	5.29	0.007–0.021	0.75–0.79
487	P30740	Leukocyte elastase inhibitor	*SERPINB1*	36	17	17	42,742	5.90	0.024	0.69
539	P01024	Complement C3c a-chain fragment	*C3*	4	6	6	39,488	4.79	0.019	0.62
1155	P04406	Glyceraldehyde-3-phosphate dehydrogenase	*GAPDH*	31	16	16	35,922	8.58	0.011	0.51
582	P40925	Malate dehydrogenase	*MDH1*	15	4	4	36,426	6.89	0.049	0.75
585, 612	P25311	Zinc-α-2-glycoprotein	*AZGP1*	38	11	11	34,259	5.58	0.019–0.024	0.57–0.63
1158	P00338	L-lactate dehydrogenase A chain	*LDHA*	30	13	11	36,558	8.46	0.0498	0.56
636	P54108	Cysteine-rich secretory protein 3	*CRISP3*	4	1	1	27,630	8,11	0.003	0.62
692	P63104	14-3-3 protein ζ/δ	*YWHAZ*	28	7	5	27,745	4.73	0.013	0.61
733	P52565	Rho GDP-dissociation inhibitor 1	*ARHGDIA*	29	6	6	23,207	5.01	0.0007	0.65
745	P52566	Rho GDP-dissociation inhibitor 2	*ARHGDIB*	44	11	11	22,988	5.08	0.007	0.53
756	P01591	Immunoglobulin J chain	*IGJ*	45	10	10	18,099	5.09	0.025	0.78
770, 771	P09211	Glutathione S-transferase P	*GSTP1*	39	7	6	23,356	5.44	0.013–0.026	0.65–0.67
1159	P12273	Prolactin inducible protein	*PIP*	69	10	10	13,523	5.40	0.032	0.80
1160	P06702	Calgranulin B	*S100A9*	32	3	3	13,241	5.71	0.027	0.70
1018	P09228	Cystatin SA	*CST2*	70	27	17	14,350	4.85	0.047	1.43
1020	P01036	Cystatin S	*CST4*	59	13	2	14,189	4.83	0.044	1.30
1162,1090,1163, 1164,1165,709, 737, 689,1166, 741, 1167	P01834	Ig κ chain C region	*IGKC*	80	8	8	11,609	6.11	0.0009–0.014	0.67–0.78
1162,1090,1163, 1164,1165,709, 737, 689,1166, 741, 1167	P0CG05, P0CG06	Ig λ chain C regions, Iso 2, 3	*IGLC2*, *IGLC3*	24	2	2	11,294 11,237	6.91 6.91	0.0009–0.014	0.67–0.78
1166, 741	P01598, P01602	Ig κ chain V-I region EU, HK102 (Fragment)	*KV106* *KV110*	32	3	2	11,788	8.49	0.0009–0.004	0.78
239, 243,246	P01876, P01877	Ig α chain C, Iso 1, 2	*IGHA1 IGHA2*	20	6	4	37,655	6.08	0.05	0.74–0.86

**Table 5:** List of identified salivary proteins found differentially expressed after the anxiety test in the odor group.

#	ID	Protein	Gene	Cov	Pep	Unic	MW (th)	pI (th)	*p*-Value (min–max)	Ratio (T1/T0) (min–max)
75, 78, 81	P01833	Polymeric immunoglobulin receptor	*PIGR*	32	26	26	83,284	5.59	0.001–0.040	0.78–0.86
170, 192	P02768	Serum albumin	*ALB*	63	56	56	69,367	5.67	0.024–0.027	0.77–0.83
216, 246, 247, 250	P01876, P01877	Ig α chain C, Iso 1, 2	*IGHA1*, *IGHA2*	16	6	5	37,655	6.08	0.001–0.031	0.74–0.86
271, 348	P0DUB6	α-amylase, 1A	*AMY1A*	55	38	38	57,768	6.34	0.003–0.038	1.16–1.21
733	P52565	Rho GDP-dissociation inhibitor 1	*ARHGDIA*	29	6	6	23,207	5.01	0.019	0.80
756, 827	P01591	Immunoglobulin J chain	*IGJ*	45	10	10	18,099	4.59	0.039	0.78
839	P30086	Phosphatidylethanolamine-binding protein 1	*PEBP1*	43	9	9	21,057	7.43	0.042	0.74
1010	Q01469	Fatty acid-binding protein, epidermal	*FABP5*	68	17	10	15,033	6.82	0.032	1.70
1047	P04080	Cystatin B	*CSTB*	52	6	4	11,139	6.96	0.026	0.66
1153	P04075	Fructose-bisphosphate aldolase A	*ALDOA*	23	11	9	39,420	8.39	0.021	0.69
706,1161, 1162, 1090,1163,1164, 1166, 741	P01834	Ig κ chain C region	*IGKC*	52	3	3	11,609	6.11	0.002–0.046	0.02–0.79
706, 1161, 1162, 1090,1163,1164, 1166, 741	P0CG05, P0CG06	Ig λchain C regions, Iso 2, 3	*IGLC2*, *IGLC3*	24	2	2	11,294	6.91	0.002–0.046	0.02–0.79
1166, 741	P01598, P01602	Ig κ chain V-I region EU, HK102 (Fragment)	*KV106*, *KV110*	30	3	2	11,788	8.49	0.022–0.046	0.02–0.04
1154	P07195	L-lactate dehydrogenase B chain	*LDHB*	22	8	6	36,639	5.72	0.0317	0.65

**Table 6:** Comparison analysis of upstream regulators predicted activated or inhibited based on z-score values (−2 ≤ z-score ≥ 2).

Upstream Regulator	Molecule Type	z-Score Control	z-Score Odor	*p*-Value	Target Molecules Control Group
methylprednisolone	chemical drug	1.784	2.646	1.1 × 10^−9^	GSTP1,HSPA1A/HSPA1B,IGHG1,IGHG2,IGHM,IGKC,IGKV1-5,IGLC2,IGLC3,PIGR,SERPINB1
lipopolysaccharide	chemical drug	−3.217	−1.124	5.25 × 10^−9^	ALB,AZGP1,C3,GSTP1,HSPA1A/HSPA1B,HSPA8,IGHG1,IGHM,IGKC,JCHAIN,LDHA,MDH1,PIGR,S100A9,SERPINB1,TF
TNF	cytokine	−1.61	−0.656	1.26 × 10^−6^	ACTB,ALB,ARHGDIB,C3,GSTP1,HSPA1A/HSPA1B,HSPA8,IGKC,LDHA,PIGR,S100A9,SERPINB1,TF
IL4	cytokine	−2.214	N/A	1.11 × 10^−5^	ACTB,ACTG1,C3,HSPA1A/HSPA1B,IGHG1,IGHG2,JCHAIN,PIGR,S100A9
β-estradiol	chemical endogenous	−1.937	−1.406	3.66 × 10^−5^	ACTB,ALB,C3,CST2,CST4,GSTP1,HSPA8,LDHA,PIGR,S100A9,TF,YWHAZ
KDM1A	enzyme	2	N/A	0.000244	ARHGDIB,AZGP1,LCP1,SERPINB1
PRDM1	transcription regulator	−1.98	N/A	0.00056	IGHG1,IGHM,JCHAIN,S100A9
IL1B	cytokine	−2.363	N/A	0.00281	C3,HSPA1A/HSPA1B,LCP1,LDHA,PIGR,S100A9
CSF2	cytokine	−1.955	N/A	0.00765	C3,LCP1,LDHA,TF
EGF	growth factor	−1.964	N/A	0.00826	GSTP1,LDHA,S100A9,TF

TNF, Tumor necrosis factor; IL4, interleukin 4; KDM1A, Lysine-specific histone demethylase 1A; IL1B, interleukin 1β; CSF2, Granulocyte-macrophage colony-stimulating factor; EGF, epidermal growth factor; GSTP1, glutathione S-transferase P; HSPA1A/HSPA1B, Heat shock 70 kDa protein1A/1B; IGHG1/IGHG2, Immunoglobulin (Ig) γ-1, 2 chain C region; IGHM, Ig µ chain C region; IGKC, Ig κ chain C region; IGKV1-5, Ig κ chain V-I region EU, HK102 (Fragment); IGLC2/IGLC3, Ig λchain C regions iso 2, 3; PIGR, Polymeric immunoglobulin receptor; SERPINB1, Leukocyte elastase inhibitor; ALB, serum albumin; AZGP1, Zinc-alpha-2-glycoprotein; C3, Complement C3c a-chain fragment; JCHAIN, Immunoglobulin J chain; LDHA, L-lactate dehydrogenase A chain; MDH1, Malate dehydrogenase; S100A9, Calgranulin B; TF, Serotransferrin; ACTB/ACTG1, Actin cytoplasmic Iso 1,2; ARHGDIB, Rho GDP-dissociation inhibitor 2; HSPA8, Heat shock cognate 71 kDa protein; CST2, Cystatin SA; CST4, Cystatin S; YWHAZ, 14-3-3 protein ζ/δ; LCP1, Plastin-2.

## Data Availability

The data used to support the findings of this study are available upon request to the authors.
